# Aspirin Use in Rheumatoid Arthritis Patients with Increased Risk of Cardiovascular Disease

**DOI:** 10.1155/2013/589807

**Published:** 2013-11-06

**Authors:** Jonida K. Cote, Androniki Bili

**Affiliations:** Rheumatology Department, Geisinger Medical Center, 100 North Academy Avenue, Danville, PA 17821, USA

## Abstract

*Objectives*. To examine the patterns of low-dose aspirin use in rheumatoid arthritis (RA) patients with high risk for coronary artery disease (CAD). *Methods*. Cross-sectional study of 36 consecutive RA patients with a Framingham score ≥10% for CAD. Eligible RA patients were provided with a questionnaire on CAD risk factors and use of low-dose aspirin. For aspirin nonusers, the reason for nonuse was requested by both the patient and rheumatologist. Questions for patients included physician's advice, self-preference, history of gastrointestinal bleeding, allergy to aspirin, or concomitant use of other anti-inflammatory medications. Questions for rheumatologists included awareness of the increased CAD risk, attribution, patient preference, history of gastrointestinal bleeding, allergy to aspirin, and medication interactions. *Results*. Patients participated in the study; 8 patients reported using daily aspirin, while 23 patients did not. The main reason cited by patients for not taking aspirin was that they were not instructed by their primary care physician (PCP) to do so (*n* = 16), which was also the main reason cited by rheumatologists (*n* = 9). *Conclusion*. This study confirmed underutilization of aspirin in RA patients at high risk for CAD, largely due to the perception that this is an issue which should be handled by the PCP.

## 1. Introduction

Rheumatoid arthritis (RA) is a chronic systemic inflammatory disease with leading cause of mortality being coronary artery disease (CAD), accounting for nearly 40–50% of deaths [1–4]. This increased burden of CAD, particularly myocardial infarction (MI), in RA is independent of traditional CAD risk factors, and it is attributed in part to chronic systemic inflammation [5, 6].

Low-dose aspirin has been shown to be beneficial for primary and secondary prevention of coronary artery disease (CAD) in numerous studies [7–10] in the general population, but this has not been studied in RA patients.

One of the most commonly used tools to calculate CAD risk in the general population is the Framingham risk score, a compilation of traditional CAD risk factors that estimates the 10-year risk of CAD risk, with risk ≥10% being the threshold for recommendation for low dose aspirin use for CAD prevention [11]. The Framingham score does not take into account RA as a risk factor for CAD, but Chung et al. showed that a higher Framingham score is independently associated with coronary artery calcification as determined by high electron beam computed tomography in RA patients [12].

To account for the increased CAD risk conferred by RA, the European League Against Rheumatism (EULAR) recommended in 2010 CAD risk score models, which are adapted for RA patients by introducing a 1.5 multiplication factor when the patient meets 2 out of 3 of the following criteria: RA disease duration for 10 years or longer, rheumatoid factor (RF) or anticyclic citrullinated peptide (CCP) positive, and presence of extra-articular manifestations [13]. 

Despite the existence of tools to calculate CAD risk in RA, there are no recommendations for the use of aspirin for primary CAD prevention in RA, and this is largely at the discretion of the treating physician. The purpose of the present study was to examine the patterns of low dose aspirin use in RA patients with a Framingham score ≥10% for CAD.

## 2. Materials and Methods

A cross-sectional study in an outpatient rheumatology clinic in Geisinger Medical Center, Danville, PA, was performed. All five rheumatology staff physicians participated. The first 36 consecutive patients with RA, defined by either the 1987 or 2010 American College of Rheumatology classification criteria [14, 15], or by a rheumatologist, seen in the rheumatology clinic from January 1, 2011 to April 30, 2013 and with a Framingham score ≥10% (http://cvdrisk.nhlbi.nih.gov/calculator.asp) were included in the study. Patients were excluded if they had preexisting CAD, diabetes mellitus (DM), long-term anticoagulation or antiplatelet therapy, gastrointestinal bleeding, or reported allergy to aspirin.

All rheumatologists' schedules were daily screened for eligible RA patients. Before the routine care visit with the rheumatologist, the patient was provided with a questionnaire regarding aspirin intake. The patient questionnaire asked for patient's gender, ethnicity, level of education, smoking status, personal, medical, or family history of hypertension, diabetes or high cholesterol, followed by daily use of aspirin. Patients who did not use aspirin were asked to provide the reason for the nonuse such as physician did not advise, self-preference, history of gastrointestinal bleeding, allergies or concomitant use of other anti-inflammatory medications, or other reason for the patient to fill in the blank.

For patients not on aspirin, a questionnaire was administered to the attending rheumatologist to describe the reasons why, in their opinion, the patient was not on aspirin. This rheumatologist questionnaire asked the rheumatologist if they were aware of high risk of CAD in RA patients, if they were aware that the eligible patient may benefit from use of low dose aspirin for primary prevention of CAD, and the reasons why the patient was not on aspirin, such as “PCP should do it”, “I did not think to prescribe it”, patient preference, history of gastrointestinal bleeding, allergy to aspirin, interaction with other medications, or other reasons for the rheumatologist to fill in the blank.

Data were manually extracted from electronic health records, including age, gender, duration of RA, extra-articular manifestations, smoking, blood pressure, DM, CAD, hyperlipidemia, total cholesterol, high density lipoprotein (HDL), rheumatoid factor (RF) positivity, anticyclic citrullinated peptide (anti-CCP) antibody positivity, corticosteroid, nonsteroidal anti-inflammatory drugs (NSAID), lipid-lowering agent, and antihypertensive medication use.

The Student's *t*-test and chi-square test that were performed were appropriate for comparison of differences between the groups of patients according to aspirin use.

This study was approved by the appropriate Geisinger Health System Institutional Research Board. All patients gave written informed consent prior to participating in the study.

## 3. Results

Patients were eligible for inclusion in the study. Five patients declined participation. The characteristics of the 31 included patients are shown in Table 1. Eight patients used low dose aspirin on a daily basis and 23 did not use aspirin. Patients who used aspirin smoked less, but were otherwise similar to the nonusers.

For the 23 patients that were not taking aspirin, the reasons for nonaspirin use are shown in Table 2(a) for patients and Table 2(b) for rheumatologists. The main reason cited by the patients for not taking aspirin was that they were not instructed by their primary care physician (PCP) to do so (*n* = 16), followed by patient preference not to take aspirin (*n* = 4). Gastrointestinal bleeding was not reported by any of the patients.

The main reason cited by rheumatologists for patients not taking aspirin was that the PCP should recommend aspirin to the patient (*n* = 9), followed by polypharmacy (*n* = 4) or patients' preference/not considering it (*n* = 3 for both). Gastrointestinal bleeding was a concern in only one patient.

## 4. Discussion

The present study suggests underutilization of aspirin in rheumatoid arthritis patients with high risk of CAD based on a Framingham risk score ≥10%. About 61% of these patients also met the EULAR criteria for cardiovascular risk management. Only 8 patients included in the study were taking aspirin. The most common reason for underutilization of aspirin was that the primary care physician (PCP) did not advise the patients to take it. This may be due to the perception that the PCP takes care of the spectrum of medical problems of the patients, as opposed to the rheumatologist who only treats the joint disease of rheumatoid arthritis. Therefore, the PCP should manage prevention issues, including CAD prevention. However, it is unknown if the PCP community is fully aware of the increased risk of CAD in RA.

There have been no other studies to look at the use of aspirin for primary CAD prevention in rheumatoid arthritis patients, but our observation concurs with studies reporting underutilization of aspirin in the general population [7].

There are no specific guidelines for the use of aspirin for CAD prevention in RA patients. With regards to CAD risk management in RA patients, the general recommendation is pursuing intervention according to the national guidelines [4, 13]. In the US, the 2002 and 2007 American Heart Association guidelines recommend aspirin for primary prevention of CAD in patients with a ten-year risk of coronary heart disease of ≥10 percent [7, 10]. The 2012 American College of Chest Physicians' guidelines suggest the use of low dose aspirin (75–100 mg daily) for persons 50 years or older without cardiovascular disease. The 2009 guidelines from the US Preventive Services Task Force encourage use of aspirin in selected populations, considering the relative cardiovascular benefit and gastrointestinal bleeding [11]. Given that the risk of CAD in RA is higher than the risk in the general population, it appears obvious to treat RA patients with aspirin for CAD prophylaxis according to the above guidelines. However, RA is also independently associated with increased risk of gastrointestinal bleeding [16, 17] and also the use of NSAIDs and corticosteroids contribute to that increased risk. In addition, use of nonsteroidal anti-inflammatory drugs (NSAIDs) such as ibuprofen and naproxen can interfere with the antiplatelet effect of aspirin [18], which may be an additional reason for not prescribing aspirin in RA patients who are more likely to use NSAIDs than the general population.

Nevertheless, fear of gastrointestinal bleeding or coadministration of NSAIDs and/or corticosteroids were not the main reasons cited for RA patients not taking aspirin. In fact, concern for gastrointestinal bleeding was only noted by one rheumatologist, and NSAID or corticosteroid use was not different between aspirin users and nonusers.

The main finding that emerged from this study is that the majority of both patients and rheumatologists view the issue of aspirin use for CAD prevention as an issue that should be handled by the PCP. It appears that there is a care gap in which the rheumatologists see the PCP as the primary owner of CAD prevention issues, but the PCP is not necessarily informed of the increased risk of CAD in RA.

In conclusion, this study showed underutilization of aspirin in RA patients at high risk for CAD, largely due to the perception that this is an issue that should be handled by the PCP. It is extremely important that the rheumatologic community dedicates its effort to educate its primary care colleagues on the higher CAD risk that RA patients carry. In addition, further discussion is needed between rheumatologists and primary care providers regarding the ownership of the care of CAD, the most devastating comorbidity of RA.

## Figures and Tables

**Table 1:** Baseline characteristics of RA patients with a Framingham score ≥10%.

Baseline patient characteristics	All patients *n* = 31	Aspirin use *n* = 8	No aspirin use *n* = 23
Age (mean)	62.2	65.5	61.0
Male gender (%)	24 (77)	6 (75)	18 (78)
Smoking (%)	12 (39)	2 (25)	10 (43)
RF+ (%)	24 (77)	7 (86)	17 (74)
RF− (%)	10 (30)	1 (13)	6 (26)
Anti-CCP+ (%)	13 (42)	3 (38)	10 (43)
Anti-CCP− (%)	7 (23)	1 (13)	6 (26)
Anti-CCP unavailable (%)	11 (35)	4 (50)	7 (30)
EULAR risk score ≥ 15 (%)	19 (61)	5 (63)	14 (61)
Prednisone use (%)	12 (39)	1 (13)	11 (48)
NSAID use (%)	11 (35)	2 (25)	9 (39)
Both prednisone and NSAID use	6 (19)	1 (13)	5 (22)

RF: rheumatoid factor; anti-CCP: anti-cyclic citrullinated peptide antibodies; EULAR: European League Against Rheumatism; NSAID: nonsteroidal anti-inflammatory drugs.

**Table:** (a) Patients' reasons

Reason	PCP did not advise	Patient preference	GI bleeding	Allergy	No reason
*n*	16	4	0	1	2

PCP: primary care physician; GI: gastrointestinal.

**Table:** (b) Rheumatologists' reasons

Reason	PCP should do it	Patient preference	Multiple medications	GI bleeding	Allergy	Hepatitis C	GI upset	No reason
*n*	9	3	4	1	1	1	1	3

PCP: primary care physician; GI: gastrointestinal.
